# The Microbiome of an Invasive Spider: Reduced Bacterial Richness, but no Indication of Microbial-Mediated Dispersal Behaviour

**DOI:** 10.1007/s00248-025-02565-6

**Published:** 2025-07-02

**Authors:** Nijat Nariman, Martin H. Entling, Henrik Krehenwinkel, Susan Kennedy

**Affiliations:** 1 Institute of Organismic and Molecular Evolution (iomE), Johannes Gutenberg-University of Mainz, Hanns-Dieter-Hüsch-Weg 15, Mainz, 55128 Germany; 2 iES Landau, Institute for Environmental Sciences, Ecosystem Analysis, University of Kaiserslautern-Landau (RPTU), Landau, 76829 Germany; 3 Department of Biogeography, Faculty of Spatial and Environmental Sciences, University of Trier, Trier, 54296 Germany

**Keywords:** Araneae, Bacterial symbionts, Dispersal, *Mermessus trilobatus*, Range expansion, 16S rRNA amplicon sequencing

## Abstract

*Mermessus* *trilobatus*, an invasive North American linyphiid spider, has expanded its invasion range up to 1400 km in Europe, accelerating its dispersal speed in less than 40 years. The high heritability of dispersal behaviour and the spatial sorting of high and low dispersers indicate a genetic basis of dispersal behaviour. However, microbial endosymbionts can moderate dispersal behaviour in related species (*Rickettsia* in *Erigone atra*). Hence, dispersal behaviour in *M. trilobatus* might also be dictated by the activity of dispersal-mediating endosymbionts. Here, we investigated the microbiome of invasive *M. trilobatus* spiders extracted from (1) high- and low-dispersive individuals and (2) spiders originating from locations close to the edge and core of the expansion. We examine the microbiomes for the presence of potential dispersal- and reproduction-mediating bacterial strains and compare the microbial assemblages of spiders based on their dispersal behaviour and locations of origin. The composition of microbial assemblages was similar among spiders of different geographic origins and dispersal behaviour. However, microbial richness was lower in high- than in low-dispersive individuals. Surprisingly, none of the known dispersal- or reproduction-altering endosymbionts of arthropods was identified in any tested spider. This contrasts with published results from North America, where *M. trilobatus* is a known host of *Rickettsia* and *Wolbachia*. Thus, the invasive European population appears to have lost its associated endosymbionts. As endosymbionts can reduce spider mobility, it is possible that their absence facilitates the spread of the invasive spider population. The absence of endosymbionts among the analysed individuals substantiates the role of genetic mechanisms behind the variable dispersal behaviour of invasive *M. trilobatus* in Europe.

**Supplementary Information:**

The online version contains supplementary material available at 10.1007/s00248-025-02565-6.

## Introduction

The introduction of invasive species to novel areas is a pressing issue of the Anthropocene, and the rate of such introductions is expected to increase rapidly [1]. Once introduced to their exotic ranges, invasive species establish and then disperse, colonising more distant areas [2, 3]. Dispersal, therefore, is a vital trait in the life history of range-expanding species [3, 4].

*Mermessus trilobatus* (Araneae: Linyphiidae), an invasive North American spider, has colonised a vast area of Europe, accelerating its expansion speed from about 150 km per decade in the 1980s to 400 km after 2010 [5, 6]. Spiders originating from areas with more recent colonisation history show twice as high dispersal propensity as their counterparts originating from the regions close to the core of the invaded range [6]. The high heritability of dispersal behaviour in *M. trilobatus* [7] and the accumulation of highly dispersive individuals in areas with the most recent spread [6] indicate a genetic background of dispersal behaviour in this invasive spider.

Nevertheless, other internal factors, such as microbial infestation, might also moderate dispersal behaviour in spiders. Notably, Goodacre and colleagues [8] have demonstrated the role of *Rickettsia* endosymbionts in the long-distance dispersal of another linyphiid species—*Erigone atra*. In their study, *Rickettsia* presence reduced dispersal propensity in *E. atra*, and antibiotic treatment increased long-distance dispersal behaviour. Further, maternally inherited bacterial symbionts such as *Cardinium*, *Rickettsia*, *Spiroplasma*, and *Wolbachia* are known to influence the reproductive ability of arthropods, including spiders, by increasing the reproductive success of infected females (e.g. [8–12]). *Rickettsia* and *Wolbachia* have already been identified in the microbiome of *M. trilobatus* individuals collected in their native range in North America (*Rickettsia* in ~ 70% and *Wolbachia* in ~ 20% of tested spiders [12]). Consequently, the dispersal behaviour and reproductive traits of *M. trilobatus* might be dictated by microbial endosymbionts, with a subsequent substantial impact on the population dynamics and spatial distribution of these spiders in Europe.

Despite the possible role of microbial infections in the life history of invasive and range-expanding species, the knowledge of host-symbiont-mediated behaviour in spiders is limited [13]. Moreover, previously unidentified microbial species are being newly described. Hence, new data on the microbiome of invasive and/or expanding species can create avenues for further investigation of host-symbiont interactions and their roles in the host’s life history.

Here, we present microbiome data of invasive *M. trilobatus* from Europe. We compare the microbial assemblages of spiders exhibiting high- and low-dispersive behaviour. Furthermore, we compare the microbial assemblages of spider offspring originating from a recently colonised area near the invasion front (since ~ 2018, Horsens, Denmark) with spiders from one long-established population close to the invasion core (since ~ 1981, Wilgartswiesen, Germany). The spiders from Horsens showed at least two times higher dispersal propensity than those originating from Wilgartswiesen [6]. We aim, thus, to address the following questions:

- Does the microbiome of *M.* *trilobatus* contain bacteria that are known to affect spider dispersal behaviour or reproduction?
- Do microbial communities differ between high- and low-dispersive spiders?
- How different are microbial communities between spiders from the edge and core of the invaded range?
- Do the microbial communities of high-dispersive and/or spiders from the invasion range edge share an unidentified bacterium absent in the microbiome of low-dispersers and/or spiders originating from the invasion core areas? Alternatively, do the low-dispersers and/or the spiders from the core areas of the invasion range harbour a unique unidentified bacterium absent in the microbiome of high-dispersers and/or spiders from the invasion range edge?

## Methods

### Spider Collection and Sample Preparation

We collected the first batch of spiders (mated females) in Landau, Germany, and Vienna, Austria, in 2020 (28 and 26 individuals, respectively) [6]. We used the first and second lab-reared generation offspring of the wild-captured females for the dispersal experiments reported elsewhere [7]. In brief, spiders were tested for dispersal behaviour three times during three consecutive days and considered high-dispersive if they demonstrated the pre-dispersal behaviour at least once daily. The spiders were considered low-dispersive if no pre-dispersal behaviour was registered during the 3 days of experiments (for more details, see [7]). For further microbiome extraction, we froze the second lab-reared generation males phenotyped as high- and low-dispersive at − 20 °C. We collected the second batch of spiders (also mated females) in two distant European locations: Horsens in Denmark and Wilgartswiesen in Germany in 2021 (16 individuals from each location). Horsens is considered close to the invasion front since all records from Denmark are from 2018 or younger. In contrast, Wilgartswiesen is situated closer to the core areas of *M. trilobatus*’ invasion range (for more details, see [6]). We then froze the first lab-reared generation male offspring from each location at − 20 °C for microbiome extraction. Note that spiders preserved in 2021 for these analyses were not phenotyped for dispersal behaviour. However, spiders from Horsens showed at least twice as high a dispersal propensity as their counterparts from Wilgartswiesen [6]. We could preserve only males because females were used for the experiments until their natural death (reported elsewhere [6, 7]).

### DNA Extraction, 16S rRNA Gene Amplicon Sequencing and Analysis

All specimen handling was carried out inside a UV workstation (PCR Workstation Pro, VWR), in which all equipment and plastics had first been irradiated under UV light for 30 min to remove any contaminating DNA. We used the whole spider specimen for DNA extraction. Each spider was surface sterilised by submerging it in an individual tube of 0.15% NaOCl for 30 min. Spiders were then transferred to individual tubes of 100% ETOH and fully submerged to remove any bleach residue. Specimens were subsequently air-dried on sterile Kimwipes before being transferred into individual tubes containing 300 µL of Cell Lysis Solution (PureGene Tissue Kit, Qiagen), 1.5 µL of Proteinase K (Qiagen), and two sterile stainless steel ball bearings (Viwanda). Forceps were sterilised in 0.5% NaOCl, then thoroughly rinsed in 100% ETOH, before and after handling each specimen. Two blank samples were included in the workflow to test for possible DNA contamination on forceps, in reagents, on Kimwipes, etc. These consisted of tubes containing only the Cell Lysis Solution, Proteinase K, and sterile ball bearings. To simulate spider handling, after having processed all of the spider specimens and re-sterilised the forceps as explained above, the tips of the forceps were dipped into a 0.15% bleach tube, then a 100% ETOH tube, then gently wiped against a sterile Kimwipe, and then swirled around inside the Cell Lysis Solution/Proteinase K tube for 5 s. These blanks were treated as samples and included in all subsequent steps. Specimens were homogenised in a 1600 MiniG tissue grinder (SPEX Sample Prep) at 1500 RPM for 3 min. They were then incubated at 55 °C for 6 h. DNA was extracted using the Qiagen PureGene kit according to the manufacturer’s protocol, with the addition of GlycoBlue Coprecipitant (ThermoFisher) in a ratio of 1:600 GlycoBlue:isopropanol, to improve the visibility of DNA pellets.

16S rRNA gene amplicon libraries were generated using the primers MS-27F and MS-338R [14]. PCR was performed using the Qiagen Multiplex kit following the manufacturer’s protocol, with 35 cycles and an annealing temperature of 55 °C. Non-template controls were included to test for contamination of the PCR reagents. The PCR primers included a 5′ Illumina tail that allowed for a second round of PCR, in which each sample was tagged with a unique combination of 8-bp F and R barcodes following Lange et al. [15], with six cycles and an annealing temperature of 56 °C. PCR success was verified by running the products on a 1.5% agarose gel. All samples, blanks, and non-template controls were pooled in equal volumes (all gel bands appeared equally strong), cleaned of residual primer using 1X AMPure beads (Beckman-Coulter), and sequenced on an Illumina MiSeq using the 500-cycle V2 paired-end sequencing kit.

Sequences were demultiplexed by index barcode combination using the MiSeq Control Software version 2.6.2.1 (Illumina). F and R reads were merged using PEAR [16] with a minimum overlap of 50 bp and a minimum quality of 20. Merged reads were then quality filtered with a minimum quality of 30 over a minimum of 90% of bases, and converted to FASTA format, using FastX-Toolkit version 0.0.13 [17]. Primer sequences were trimmed off using awk [18] and sed. Trimmed FASTA files were dereplicated and denoised into zero-radius operational taxonomic units (zOTUs) using USEARCH version 10.0.240 [19]. zOTUs were taxonomically identified by BLAST searching [20] against the NCBI nucleotide database and annotated using blast2taxonomy version 1.3.4 [21]. DNA extraction blanks and non-template PCR controls were used to check for contaminants in the data: all zOTUs that recovered at least two reads in any of these controls were removed from the data set. To address possible index bleeding or carryover, all read numbers lower than ten were deleted and replaced with zeros using Find/Replace in Microsoft Excel version 16051.14430.20306.0. After this cleaning step, some of the samples (spiders) and bacterial zOTUs returned only zero values. We removed these individuals and zOTUs from subsequent analysis (see Table 1 for the sample sizes).

**Table 1:** Sample sizes of spiders from which microbiomes were extracted and analysed, as well as the mean number of bacterial OTUs (alpha diversity, richness) observed in each group. The number of spiders used for the analysis is shown in brackets

Group/origin	Number of spiders	Mean number of bacterial OTUs	*P* value
High-dispersive	19 (15)	7.5	0.0032
Low-dispersive	21 (21)	12	
Horsens	40 (39)	10.5	0.5701
Wilgartswiesen	31 (31)	9.8	

All read numbers lower than ten were deleted and replaced with zeros to tackle possible index bleeding or carryover. Hence, some of the samples (spiders) and bacterial zOTUs returned only zero values and were removed from subsequent analysis

All analyses were performed in R (version 4.4.1; [22]) through RStudio (version 2024.09.0; [23]) using the package *vegan* [24] unless specified otherwise. Due to complete independence (different sets of spiders, sampling years, and generations), we analysed the data separately based on spiders’ dispersal behaviour (high vs low) and origin (Horsens vs Wilgartswiesen). We used zOTU relative abundance (read numbers) for all analyses.

First, we calculated the within-sample diversity (alpha) for high- and low-dispersive spiders as well as based on the origin of spiders (*estimateR* function) using the number of observed bacterial OTUs as a proxy for richness. We then compared the richness of microbial communities between high- and low-dispersive spiders and between spiders originating from Horsens (invasion front) and Wilgartswiesen (invasion core) training negative binomial generalized linear models (*glm.nb* function from package *MASS*; [25]). The negative binomial distribution was selected because it is the most flexible and suitable for count data [26]. We then applied the ANOVA *χ*^2^-test (*ANOVA* function in package *car*; [27]) to the *glm.nb* models.

For between-sample diversity (beta), we first standardised the data using *Hellinger* transformation (*decostand* function). We then calculated Bray–Curtis dissimilarity indices (*vegdist* function) as a quantitative measure of microbial beta diversity among spiders with different dispersal behaviour and origins. We used these values to create non-metric multidimensional scaling ordinations (NMDS; *metaMDS* function) to evaluate the roles of spiders’ dispersal behaviour and origin. We calculated PERMANOVA tests (*adonis2* function) on these dissimilarity indices to test for significant differences. The plots were created with *ggplot2* [28], *viridis* [29], and *rgl* [30] packages.

## Results

In total, we identified 121 microbial OTUs (25,157 reads in total) in high- and low-dispersive spiders and 218 (57,883 reads in total) in spiders originating from two distant locations (Horsens and Wilgartswiesen). No *Rickettsia* strains were identified in any spider, indicating that dispersal behaviour in this species is not influenced by the presence of hitherto known dispersal-mediating bacterial symbionts. Interestingly, none of the most common endosymbionts of spiders, namely *Cardinium*, *Spiroplasma*, and *Wolbachia*, was found in any tested spider. The microbiomes of most of the spiders were dominated by *Renibacterium salmoninarum* and *Mycobacteroides abscessus* (44% of all reads; Table 2). Furthermore, no unique unidentified bacterium was present in the microbiome of only high-dispersive spiders and/or spiders originating from the invasion range edge (Horsens) and absent in low-dispersers and/or spiders from the invasion core population (Wilgartswiesen). Similarly, microbiomes of low-dispersers and/or spiders from the core of the invasion range (Wilgartswiesen) harboured no unidentified bacterium absent in high-dispersive spiders and/or spiders originating from the invasion range edge (Horsens).

**Table 2:** The total and average number of reads of the two most common OTUs identified in the microbiome of most of the 111 tested *M. trilobatus* individuals (44% of all reads)

Strain	Total number of reads	Average number of reads	Number of host spiders (% spiders)
*Renibacterium salmoninarum*	19,387	175	69 (62%)
*Mycobacteroides abscessus*	17,432	157	76 (68%)

**Fig. 1:**
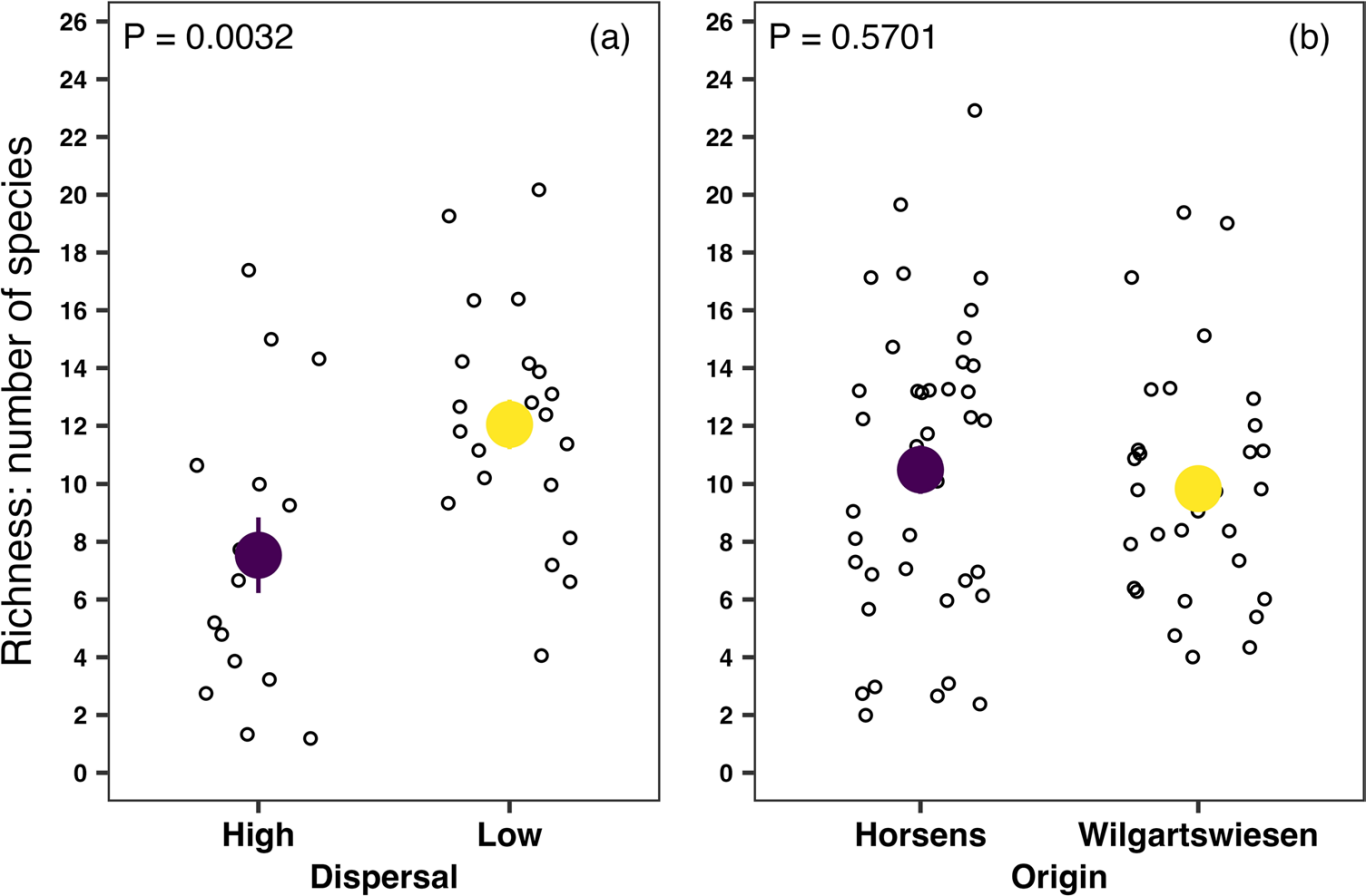
The richness of microbial OTUs identified in the **a** high- and low-dispersive spiders and **b** spiders originating from two distant locations in Europe (Horsens in Denmark and Wilgartswiesen in Germany) using the number of observed OTUs as a proxy of alpha diversity

On average, high-dispersive spiders harboured around 40% fewer bacterial OTUs (richness; alpha diversity) than their low-dispersive counterparts (7.5 vs 12, respectively; *χ*^2^ = 8.7, *P* = 0.0032; Fig. 1a; Table 1). Analyses of alpha diversity based on the spiders’ origin indicated no significant differences in microbial richness between Horsens and Wilgartswiesen spiders (10.5 vs 9.8, respectively; *χ*^2^ = 0.3, *P* = 0.5701; Fig. 1b; Table 1).

**Fig. 2:**
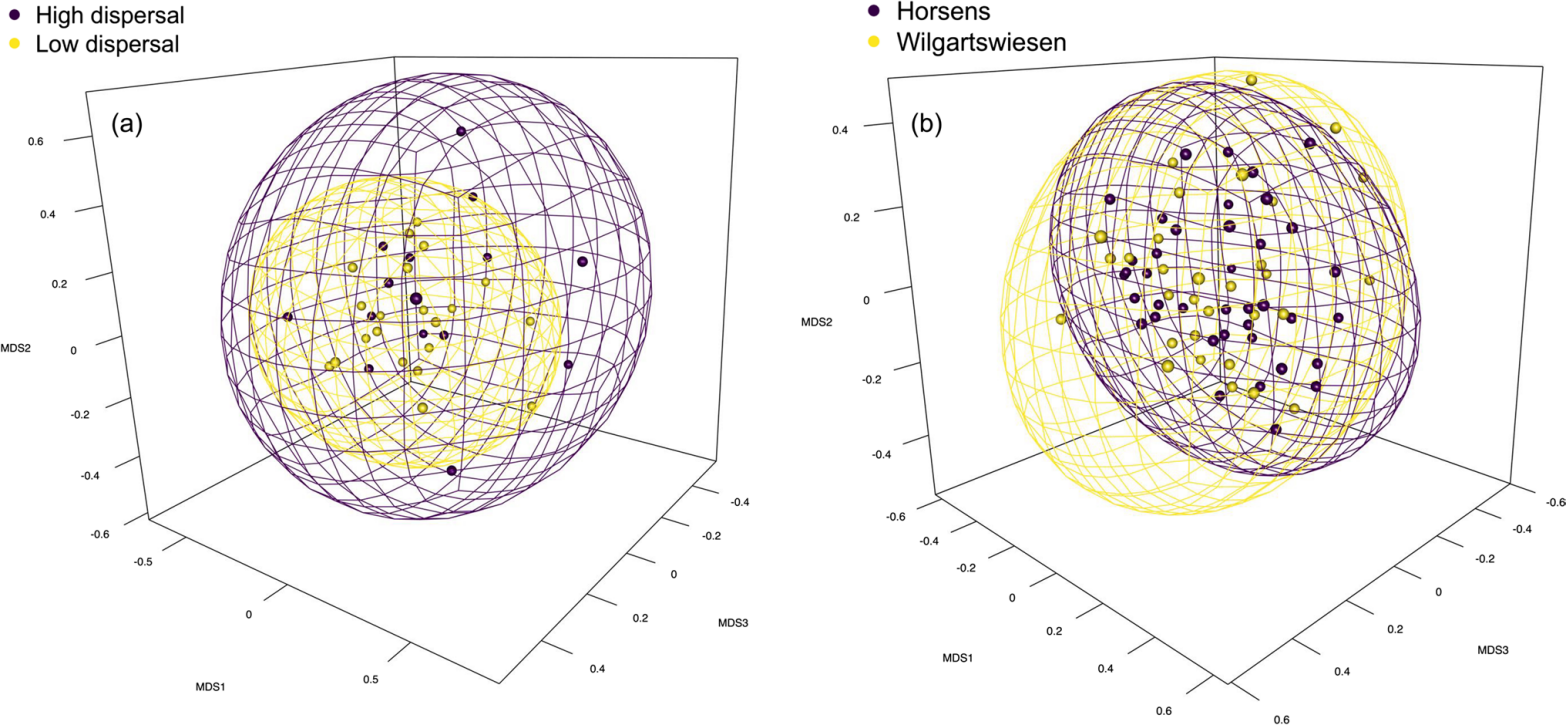
The ordination plots display non-metric multidimensional scaling (NMDS) on dissimilarities of **a** high- and low-dispersive spiders (stress value = 0.17) and **b** spiders originating from Horsens and Wilgartswiesen (stress value = 0.18). See Figs. S1 and S2 in the supplementary information for the video animations

The ordination with NMDS revealed no visible spatial difference in microbial communities between spiders with high- and low-dispersive behaviours (*k* = 3; stress = 0.17; Fig. 2a) and based on spiders’ origin (*k* = 3; stress = 0.18; Fig. 2b). These similarities were also supported statistically both based on spiders’ dispersal (PERMANOVA; *F*_(1,34)_ = 1.3; *P* = 0.141) and their origin (PERMANOVA; *F*_(1,68)_ = 0.9; *P* = 0.463), indicating no significant community differences between compared groups. We also analysed the presence/absence data for validation, which yielded similar results (dispersal: PERMANOVA; *F*_(1,34)_ = 1.0; *P* = 0.488; origin: PERMANOVA; *F*_(1,68)_ = 1.2; *P* = 0.245).

## Discussion

The microbiome of high- and low-dispersive spiders, as well as spiders originating from the edge and core of the expansion range of *M. trilobatus*, harboured no microbial strain previously known to influence the dispersal behaviour in spiders (*Rickettsia*; [8]), supporting the role of genetic background and not microbial infections in the dispersal behaviour of *M. trilobatus*. Furthermore, none of the reproduction-mediating endosymbionts in other arthropods (e.g. *Rickettsia*, *Rickettsiella*,* Wolbachia*; [31–33]) has been identified in any analysed spider. Overall, the microbial assemblages of the tested *M. trilobatus* spiders showed high similarities based on the dispersal behaviour (high vs low) and originating locations (core vs edge of the expansion range). Interestingly, unlike their native conspecifics in North America [12] and many arthropods [10, 34], invasive *M. trilobatus* individuals in Europe did not carry any of the most common microbial endosymbionts. This fact suggests that the absence of endosymbionts contributes to a generally high mobility of the European invasive population compared to native counterparts in North America. Even if no role of microbial infections was detected behind the current variation of dispersal propensity, the absence of endosymbionts could have facilitated the spread of *Mermessus* at an early invasion stage. It could even be speculated that the loss of *Rickettsia* infections in some *Mermessus* individuals of the European founder population has enhanced their mobility, giving them a selective advantage in the new range with ample unoccupied habitat. Such a mechanism provides a possible explanation for the loss of dispersal-suppressing endosymbionts also in other invasive species.

Surprisingly, European invasive spiders seem to lack the most common endosymbionts of spiders (e.g. *Cardinium*, *Spiroplasma*, *Wolbachia*, *Rickettsia*; [10, 12]). However, the native *M. trilobatus* in the USA hosts *Rickettsia* and *Wolbachia* [12]. The loss of facultative endosymbionts has been previously reported for invasive species (e.g. Green Peach Aphid; [39]). Possible explanations include environmental mismatch [40], increased immune response [35, 36, 41], associated high energy costs [42], or founder effects of the invasive populations. Furthermore, a sister species of *M. trilobatus*—*M. fradeorum*—harbours *Rickettsia*, *Rickettsiella*, and *Wolbachia* as endosymbionts in North America, and laboratory experiments prove their significant role in hosts’ feminisation and cytoplasmic incompatibility [31, 32] as well as possible temperature dependence of *Rickettsiella*-driven cytoplasmic incompatibility [43]. Working with *M. trilobatus* since 2019, we have never observed feminisation among the lab-reared European populations (NN and ME personal observations). Nevertheless, the possibility that a loss of endosymbionts is typical in invasive versus native populations needs thorough investigation.

Independent of the absence of endosymbionts, high-dispersive spiders hosted a substantially lower number of microbial strains than low-dispersive individuals. This trend might be a surprise since vagile individuals should be exposed to more microbial infections than their less mobile counterparts due to the frequent environmental changes they face. However, high dispersal ability is often coupled with a strong immune response (e.g. [35, 36], but see [37]), which could translate into the lower bacterial richness of the microbiome of vagile versus sessile individuals, especially in the lab-reared individuals that did not have an opportunity to expose themselves to different environments. Similarly, compared to specialist predators, generalists have a higher tolerance to entomopathogenic bacteria associated with a more efficient cell-mediated immune response, most likely due to broader pathogen exposure rates of generalist versus specialist insect species [38].

The relations between the invasive spiders and the two most abundant microbial species identified in tested spiders, namely *Renibacterium salmoninarum* and *Mycobacteroides abscessus*, remain unclear due to a lack of knowledge of the relation between these two bacteria and arthropods, especially spiders. *Renibacterium* *salmoninarum* is notorious for causing bacterial kidney disease worldwide, particularly in salmonid fish [44]. *Mycobacteroides abscessus* is known as a human pathogen in the medical community [45]. To our knowledge, the associations between *R. salmoninarum*, *M. abscessus*, and spiders have never been studied. However, *M. abscessus* has been isolated from natural and artificial water sources and can easily survive in soil [45] from where this bacterium, as well as *R. salmoninarum*, can find their way into spiders’ microbiome through the soil arthropods on which spiders feed. *Renibacterium* *salmoninarum*, in particular, has already been identified in the microbiome of ticks [46]. Nevertheless, the associations between these two bacteria and spiders must be further investigated.

One limitation of our study is that all microbiomes are from male individuals. Nevertheless, males of *M. trilobatus* disperse more often than females [6, 7] and, hence, represent ideal study systems for investigating the link between microbial assemblage and dispersal behaviour. Further, we extracted the microbiome of only lab-reared spiders, which could explain our dataset’s overall low read number. A significant reduction of certain microbial strains in the biome of lab-reared individuals can be expected after 3 and a complete disappearance only after 6 months of lab-rearing in mites [47]. In contrast, tested spiders here were bred in the lab for only around 1 (one generation; Horsens and Wilgartswiesen spiders) and up to 2 months (two generations; high- and low-dispersive spiders). Thus, we assume that endosymbionts would have been detected if they had been present in the sampled wild populations. Notably, the differences in dispersal propensity between populations [6] and heritability experiments [7] were assessed with lab-reared first and second generations of *M. trilobatus*, respectively. Thus, the absence of behaviour-mediating endosymbionts in the current study suggests that they were equally absent in the individuals used in the above studies. This substantiates that inherited differences in dispersal behaviour previously observed in *M. trilobatus* [6, 7] are genetic and unrelated to the possible transfer of microbial infections across generations.

In conclusion, the high similarity of microbial assemblages of high- and low-dispersiveal spiders, as well as microbiome assemblages of spiders originating from distant locations within the invaded range, indicates no impact of the microbiome on dispersal behaviour in the European invasive population of *M. trilobatus*. This is underlined by the absence of *Rickettsia* or other known behaviour-mediating endosymbionts in any tested spiders. Consequently, variation in dispersal behaviour in invasive *M. trilobatus* is most likely based on molecular mechanisms, which are yet to be investigated. Furthermore, invasive populations of *M. trilobatus* in Europe seem to have lost the endosymbionts that are common in most arthropods and also in their native populations in North America. Highly dispersive individuals harbour reduced bacterial richness compared to low dispersers, possibly due to the more potent immune response of high- versus low-dispersive spiders. Hence, dispersal behaviour may drive bacterial endosymbiont assemblage, rather than bacterial assemblage driving dispersal, in this invasive spider.

## Supplementary Information

Below is the link to the electronic supplementary material.

ESM1 (PDF 150 KB)

## Data Availability

Data generated and analysed during this study are available from Figshare: 10.6084/m9.figshare.28504874.v1.

## References

[1] H Seebens S Bacher TM Blackburn C Capinha W Dawson S Dullinger P Genovesi PE Hulme M Kleunen Van I Kühn JM Jeschke B Lenzner AM Liebhold Z Pattison J Pergl P Pyšek M Winter F Essl 2021 Projecting the continental accumulation of alien species through to 2050 Glob Change Biol 27 970 982 10.1111/gcb.15333 10.1111/gcb.15333 33000893

[2] TM Blackburn P Pyšek S Bacher JT Carlton RP Duncan V Jarošík JRU Wilson DM Richardson 2011 A proposed unified framework for biological invasions Trends Ecol Evol 26 333 339 10.1016/j.tree.2011.03.023 21601306 10.1016/j.tree.2011.03.023

[3] A Chuang CR Peterson 2016 Expanding population edges: theories, traits, and trade-offs Glob Change Biol 22 494 512 10.1111/gcb.13107 10.1111/gcb.13107 26426311

[4] D Bonte M Dahirel 2017 Dispersal: a central and independent trait in life history Oikos 126 472 479 10.1111/oik.03801

[5] Dumpert K, Platen R (1985) Zur Biologie eines Buchenwaldbodens. 4. Die Spinnenfauna. Carolinea 42:75–106. https://www.zobodat.at/pdf/Carolinea_42_0075-0106.pdf

[6] N Narimanov T Bauer D Bonte L Fahse MH Entling 2022 Accelerated invasion through the evolution of dispersal behaviour Glob Ecol Biogeogr 31 2423 2436 10.1111/geb.13599

[7] N Narimanov D Bonte MH Entling 2022 Heritability of dispersal in a rapidly spreading invasive spider Anim Behav 183 93 101 10.1016/j.anbehav.2021.11.002

[8] SL Goodacre OY Martin D Bonte L Hutchings C Woolley K Ibrahim C George Thomas GM Hewitt 2009 Microbial modification of host long-distance dispersal capacity BMC Biol 7 32 10.1186/1741-7007-7-32 19545353 10.1186/1741-7007-7-32 PMC2706808

[9] S Charlat GDD Hurst H Merçot 2003 Evolutionary consequences of Wolbachia infections TiG 19 217 223 10.1016/S0168-9525(03)00024-6 12683975 10.1016/S0168-9525(03)00024-6

[10] Goodacre SL, Martin OY (2013) Endosymbiont infections in spiders. In: Nentwig W (ed) Spider ecophysiology. Springer Berlin Heidelberg, pp 93–105. 10.1007/978-3-642-33989-9_8

[11] SJ Perlman MS Hunter E Zchori-Fein 2006 The emerging diversity of *Rickettsia* Proc Biol Sci 273 2097 2106 10.1098/rspb.2006.3541 16901827 10.1098/rspb.2006.3541 PMC1635513

[12] JA White A Styer LC Rosenwald MM Curry KD Welch KJ Athey EG Chapman 2020 Endosymbiotic bacteria are prevalent and diverse in agricultural spiders Microb Ecol 79 472 481 10.1007/s00248-019-01411-w 31300838 10.1007/s00248-019-01411-w

[13] ES Durkin ST Cassidy R Gilbert EA Richardson AM Roth S Shablin CN Keiser 2021 Parasites of spiders: their impacts on host behaviour and ecology JoA 49 281 298 10.1636/JoA-S-20-087

[14] MS Donia WF Fricke F Partensky J Cox SI Elshahawi JR White AM Phillippy MC Schatz J Piel MG Haygood J Ravel 2011 Complex microbiome underlying secondary and primary metabolism in the tunicate-*Prochloron* symbiosis PNAS 108 E1423 E1432 10.1073/pnas.1111712108 22123943 10.1073/pnas.1111712108 PMC3251135

[15] V Lange I Böhme J Hofmann K Lang J Sauter B Schöne P Paul V Albrecht JM Andreas DM Baier J Nething 2014 Cost-efficient high-throughput HLA typing by MiSeq amplicon sequencing BMC Genomics 15 1 11 10.1186/1471-2164-15-63 24460756 10.1186/1471-2164-15-63 PMC3909933

[16] J Zhang K Kobert T Flouri A Stamatakis 2014 PEAR: a fast and accurate Illumina paired-end reAd mergeR Bioinformatics 30 614 620 10.1093/bioinformatics/btt593 24142950 10.1093/bioinformatics/btt593 PMC3933873

[17] Gordon A, Hannon G (2010) Fastx-toolkit. FASTQ/A short-reads preprocessing tools (unpublished) Retrieved from http://hannonlab.cshl.edu/fastx_toolkit

[18] AV Aho BW Kernighan PJ Weinberger 1979 Awk—a pattern scanning and processing language Softw Pract Exper 9 267 279 10.1002/spe.4380090403

[19] RC Edgar 2010 Search and clustering orders of magnitude faster than BLAST Bioinformatics 26 2460 2461 10.1093/bioinformatics/btq461 20709691 10.1093/bioinformatics/btq461

[20] SF Altschul W Gish W Miller EW Myers DJ Lipman 1990 Basic local alignment search tool J Mol Biol 215 403 410 10.1016/S0022-2836(05)80360-2 2231712 10.1016/S0022-2836(05)80360-2

[21] Schöneberg Y (2023) “yschoeneberg/blast2taxonomy: v1.3.4”. 10.5281/zenodo.10009721

[22] R Core Team (2024) R: a language and environment for statistical computing. R Foundation for Statistical Computing, Vienna, Austria. Version 4.4.1, Race for Your Life. URL https://www.R-project.org/

[23] Posit team (2024). RStudio: integrated development environment for R. Posit Software, PBC, Boston, MA. http://www.posit.co/

[24] Oksanen J, Simpson G, Blanchet F, Kindt R, Legendre P, Minchin P, O’Hara R, Solymos P, Stevens M, Szoecs E, Wagner H, Barbour M, Bedward M, Bolker B, Borcard D, Carvalho G, Chirico M, De Caceres M, Durand S, Evangelista H, FitzJohn R, Friendly M, Furneaux B, Hannigan G, Hill M, Lahti L, McGlinn D, Ouellette M, Ribeiro Cunha E, Smith T, Stier A, Ter Braak C, Weedon J (2024) vegan: community ecology package. R package version 2.6–8, https://CRAN.R-project.org/package=vegan

[25] WN Venables BD Ripley 2002 Modern applied statistics with S 4 Springer New York

[26] RB O’Hara DJ Kotze 2010 Do not log-transform count data Methods Ecol Evol 1 118 122 https://doi.org/10.1111/j.2041-210X.2010.00021.x@10.1111/(ISSN)2041-210X.TOPMETHODS

[27] Fox J, Weisberg S (2019) An R companion to applied regression, 3rd edn. Sage, Thousand Oaks CA. https://www.john-fox.ca/Companion

[28] H Wickham 2016 ggplot2: elegant graphics for data analysis Springer-Verlag New York

[29] Garnier S, Ross N, Rudis R, Camargo AP, Sciaini M, Scherer C (2024) viridis(Lite) - colorblind-friendly color maps for R. viridis package version 0.6.5. https://cran.r-project.org/package=viridisLite

[30] Murdoch D, Adler D (2024) rgl: 3D visualization using OpenGL. R package version 1.3.1. https://CRAN.R-project.org/package=rgl

[31] MM Curry LV Paliulis KD Welch JD Harwood JA White 2015 Multiple endosymbiont infections and reproductive manipulations in a linyphiid spider population Heredity 115 146 152 10.1038/hdy.2015.2 25899011 10.1038/hdy.2015.2 PMC4815443

[32] LC Rosenwald MI Sitvarin JA White 2020 Endosymbiotic *Rickettsiella* causes cytoplasmic incompatibility in a spider host Proc Biol Sci 287 20201107 10.1098/rspb.2020.1107 32635864 10.1098/rspb.2020.1107 PMC7423472

[33] N Wybouw F Mortier D Bonte 2022 Interacting host modifier systems control *Wolbachia*-induced cytoplasmic incompatibility in a haplodiploid mite Evol Letters 6 255 265 10.1002/evl3.282 10.1002/evl3.282 PMC9233175 35784453

[34] Y Kikuchi 2009 Endosymbiotic bacteria in insects: their diversity and culturability M&E 24 195 204 10.1264/jsme2.ME09140S 10.1264/jsme2.me09140s 21566374

[35] Møller AP, Martín‐Vivaldi M, Soler JJ (2004) Parasitism, host immune defence and dispersal. J Evol Biol 3:603–612. 10.1111/j.1420-9101.2004.00694.x 10.1111/j.1420-9101.2004.00694.x 15149403

[36] C Romeo J Filipe LA Wauters S Comazzi F Riva N Ferrari 2023 Shifts in immune responses of an invasive alien species: a test of the evolution of increased competitive ability hypothesis using American Eastern gray squirrels in Italy Sci Total Environ 900 16574710.1016/j.scitotenv.2023.165747 37495139 10.1016/j.scitotenv.2023.165747

[37] GP Brown R Shine 2014 Immune response varies with rate of dispersal in invasive cane toads (*Rhinella marina*) PLoS ONE 9 e9973410.1371/journal.pone.0099734 24936876 10.1371/journal.pone.0099734 PMC4061023

[38] A Barthel I Kopka H Vogel P Zipfel DG Heckel AT Groot 2014 Immune defence strategies of generalist and specialist insect herbivores Proc Biol Sci 281 20140897 10.1098/rspb.2014.0897 24943370 10.1098/rspb.2014.0897 PMC4083799

[39] Q Yang PA Umina S Wei C Bass W Yu KL Robinson A Gill D Zhan SE Ward A Rooyen Van AA Hoffmann 2023 Diversity and regional variation of endosymbionts in the green peach aphid, *Myzus persicae* (Sulzer) Diversity 15 206 10.3390/d15020206

[40] H Li Z Jiang J Zhou X Liu Y Zhang D Chu 2023 Ecological factors associated with the distribution of *Bemisia tabaci* cryptic species and their facultative endosymbionts Insects 14 252 10.3390/insects14030252 36975937 10.3390/insects14030252 PMC10053707

[41] A Felden JW Baty DG Chapple MAM Gruber J Haywood C Paris AV Suarez ND Tsutsui PJ Lester 2024 Variable viral loads and immune response in an invasive ant’s native and introduced ranges Divers Distrib 30 e1386710.1111/ddi.13867

[42] C Clavé A Sugio S Morlière S Pincebourde J-C Simon V Foray 2022 Physiological costs of facultative endosymbionts in aphids assessed from energy metabolism Fun Ecol 36 2580 2592 10.1111/1365-2435.14157

[43] JD Proctor V Mackevicius-Dubickaja Y Gottlieb JA White 2024 Warm temperature inhibits cytoplasmic incompatibility induced by endosymbiotic *Rickettsiella* in spider hosts Environ Microbiol 26 e1669710.1111/1462-2920.16697 39253751 10.1111/1462-2920.16697

[44] Bayliss SC, Verner-Jeffreys DW, Ryder D, Suarez R, Ramirez R, Romero J, Pascoe B, Sheppard SK, Godoy M, Feil EJ (2018) Genomic epidemiology of the commercially important pathogen *Renibacterium salmoninarum* within the Chilean salmon industry. Microb Genom 4. 10.1099/mgen.0.000201 10.1099/mgen.0.000201 PMC6202448 30040063

[45] KC Ferrell MD Johansen JA Triccas C Counoupas 2022 Virulence mechanisms of *Mycobacterium abscessus*: current knowledge and implications for vaccine design Front Microbiol 13 84201710.3389/fmicb.2022.842017 35308378 10.3389/fmicb.2022.842017 PMC8928063

[46] A Murrell SJ Dobson X Yang E Lacey SC Barker 2003 A survey of bacterial diversity in ticks, lice and fleas from Australia Parasitol Res 89 326 334 10.1007/s00436-002-0722-4 12632173 10.1007/s00436-002-0722-4

[47] F Zélé I Santos M Matos M Weill F Vavre S Magalhães 2020 Endosymbiont diversity in natural populations of *Tetranychus mites* is rapidly lost under laboratory conditions Heredity 124 603 617 10.1038/s41437-020-0297-9 32047292 10.1038/s41437-020-0297-9 PMC7080723

