# Supplementary material for: The Microbiome of an Invasive Spider: Reduced Bacterial Richness, but no Indication of Microbial-Mediated Dispersal Behaviour
Source: Microb Ecol. 2025 Jul 2;88(1):70. doi: 10.1007/s00248-025-02565-6 (PMC12222337; doi:10.1007/s00248-025-02565-6)
Supplement: Supplementary file 1 — (PDF 150 KB) [file 248_2025_2565_MOESM1_ESM.pdf]

SUPPLEMENTAL INFORMATION

The microbiome of an invasive spider: Reduced bacterial richness, but no indication of microbial-mediated dispersal behaviour

Nijat Nariman *, Martin H. Entling, Henrik Krehenwinkel and Susan Kennedy

* Correspondence and present address: Nijat Nariman; Institute of Organismic and Molecular Evolution (iomE), Johannes Gutenberg-University of Mainz, Hanns-Dieter-Hüsch-Weg 15, Mainz, 55128, Germany. Email: nijat.nariman@gmail.com / nnariman@uni-mainz.de; ORCID: 0000-0003-1321-3243.

Link to *Figshare*: https://doi.org/10.6084/m9.figshare.28504835.v1

Figure S1

The 3D ordination animation displays non-metric multidimensional scaling (NMDS) on Bray-Curtis dissimilarities of high- and low-dispersive invasive *Mermessus trilobatus* (stress value = 0.17).

Figure S2

The 3D ordination animation displays non-metric multidimensional scaling (NMDS) on Bray-Curtis dissimilarities of invasive *Mermessus trilobatus* spiders from Horsens in Denmark (location close to the edge of the expansion range, present since ~2018) and Wilgartswiesen in Germany (location close to the core of the expansion range, present since ~1981; stress value = 0.18).
